# The diagnostic MRCP examination: overcoming technical challenges to ensure clinical success

**DOI:** 10.2349/biij.4.4.e28

**Published:** 2008-10-01

**Authors:** G Mandarano, J Sim

**Affiliations:** School of Medical Sciences, Medical Radiations, RMIT University, Victoria, Australia

**Keywords:** MRCP, MRI, sequences, ERCP, radiographer, hydrography

## Abstract

The magnetic resonance cholangiopancreatography (MRCP) examination has all but replaced the diagnostic endoscopic retrograde cholangiopancreatography (ERCP) examination for imaging the biliary tree and pancreatic ducts in many practical aspects of the clinical setting. Despite this increase in popularity, many magnetic resonance imaging (MRI) radiographers still find aspects of the MRCP examination quite challenging. The aim of this tutorial paper is to provide useful technical advice on how to overcome such perceived challenges and thus produce a successful diagnostic MRCP examination. This paper will be of interest to novice MRI radiographers who are at the beginning of their learning curve in MRCP examination. Other MRI radiographers who are interested in practical tips for protocol variations may also find the paper useful.

## INTRODUCTION

A comprehensive diagnostic magnetic resonance cholangiopancreatography (MRCP) examination should provide maximum information pertaining to the hepatic, biliary and pancreatic ducts. Common protocols of the MRCP examination include heavily T2-weighted sequences [1-8], acquired either with thin slice sections or thick slabs or both [9-12]. As the inherent biliary fluid is used as a contrasting mechanism, the broad new term of magnetic resonance hydrography has been coined in recent years [13-15]. However, if a patient has been scheduled for an MRCP examination, which may usually last for thirty minutes, it would be wise to include a few dedicated (initially, non-intravenous contrast media enhanced) magnetic resonance imaging (MRI) sequences to evaluate the contents of the upper abdomen, essentially the pancreas and the liver. Some authors [2, 5, 8, 16-20] use the acronyms MRCP and MRI to emphasise these separate and distinct aspects of imaging [4]. The rationale behind acquiring images of the pancreas and liver is to exclude the presence of any pathology associated with these organs that may affect the calibre or condition of any ducts [2,5, 6-7, 16-17,18,21,]. This is because pathology of these organs may manifest itself clinically as duct disease or can directly impinge upon, and affect, these ducts [3].

Since its clinical introduction well over a decade ago [1], the MRCP/MRI examination has played at least two very important roles. Firstly it has provided both clinicians and patients with a highly accurate diagnostic test to assess the ducts (hepatobiliary and pancreatic) and associated organs (liver, gall bladder, pancreas) [16]. The MRCP/MRI examination is non-invasive [8,9, 20, 22, 23] and less costly [12] than the diagnostic aspect of an endoscopic retrograde cholangiopancreatography (ERCP). Comparatively, the MRCP/MRI requires less examination time, fewer staff, and involves no ionising radiation [12]. In addition, the ERCP has associated morbidity and mortality rates, albeit relatively low [4, 24-26]. From the latter arises the second important role played by the MRCP/MRI examination: it has allowed more accurate selection of patients who would benefit from surgery [16, 18] and/or the therapeutic component of the ERCP. Thus it has prevented patients from undergoing an unnecessary invasive diagnostic ERCP procedure [22]. Aside from enhancing patient safety, MRCP/MRI has resulted in saving staff time, material resources and finance in the long-term [22] (although to date, there has not been a definitive agreed-upon dollar value published, or a formula that clinical centres can use to calculate such savings). This should lead to improved use, and allocation of assets and resources can therefore be directed to more urgent areas of clinical practice.

This tutorial paper provides a synopsis of current practice and emerging trends. It describes the pulse sequences commonly adopted in a MRCP examination, with the focus on the rationale of the protocol as well as practical suggestions for MRI radiographers in ensuring a successful examination.

## REVIEW OF RELEVANT ANATOMY

Understanding and appreciating the complexity of anatomy is an essential factor to the success of any MRI examination. The reader is referred to any reputable text to review the anatomy of the hepato-biliary system.

## TECHNICAL CONSIDERATIONS PATIENT PREPARATION AND INSTRUCTIONS

To ensure that the gall bladder, hepatobiliary and pancreatic ducts are filled with fluid and at their maximum distension, the patient would need to fast. It is recommended that the patient be nil per oral for at least four hours prior to commencing the examination [2, 4, 10]. Throughout this period, the patient is permitted to drink clear fluids only (namely water), and routine medication is allowed as per normal.

When the patient arrives for their appointment, the radiographer must follow the centre’s policy in relation to safety screening and this can vary from one centre to another; however all reasonable precautions must be taken to ensure that the patient is safe to enter the MRI environment.

The next important step is to instruct the patient on the specific breathing instructions and inform the patient that they will hear the radiographer’s voice through their headphone or speaker prompting them when to suspend expiration. The authors strongly believe that clear explanation of breathing instructions is a crucial step that determines the overall success or failure of the examination. This is because the main pancreatic duct is very susceptible to respiratory motion [6], as suspended respiration is more consistent for the patient to perform rather than suspended inspiration. Thus, it is advisable to practice the respiratory motion with the patient at this point. In the authors’ opinion, the approach which seems to be most successful is to instruct the patient to “breathe in, breathe out, breathe in and breathe out, and stop.” Inform them that they are expected to suspend expiration for approximately fifteen seconds and that the hyperventilation breathing should allow them to fill their lungs with air to comfortably sustain the period of suspended expiration. Mitchell [32] also concurs that suspended expiration is more consistent, and provides less motion variation, whereas full inspiration should be reserved for situations where the lung diaphragm needs to be in a more inferior position. It is imperative that the patient understands their role and that their co–operation and active participation is needed to ensure overall diagnostic success. If the breath hold technique is not adequate, then the CBD and the main pancreatic duct may not appear to unite or may appear either stenotic or dilated [33].

The next critical component is positioning of the patient, the respiratory bellows and the imaging coils. At this point, the adult patient should be lying supine on the MRI table positioned appropriately over the posterior half of the body array coil and also such that their feet will be entering the bore of the magnet first. To position the respiratory bellows correctly, the radiographer must first observe the rise and fall of the patient’s chest and abdomen with their breathing [34]. It is wise to repeat the breathing instructions while observing the patient’s chest and abdomen. The respiratory bellows need to be positioned across the point where the maximum difference in rise and fall occurs. Once the respiratory bellows are positioned, the radiographer must then observe the respiratory waveform that appears on the operator’s console. It must display the distinct rise and fall wave patterns and these patterns need to be regular. If the patient has breathing difficulties or can only take shallow breaths, one method to ensure a respiratory wave pattern is to place the respiratory bellows *diagonally* across the region (either the chest or the abdomen) that corresponds with the patient’s breathing. From the authors’ clinical experience, this will ensure that any respiratory motion will be detected. However, when the respiratory pattern from this technique is observed on the console monitor, it may only display shallow peaks.

Next, pads or sponges are placed alongside the respiratory bellows. These prevent the respiratory bellows from being compressed by the weight of the anterior half of the imaging coils. If the respiratory bellows were to be compressed they would be unable to detect the patient’s respiratory motion or may not accurately represent the respiratory waveform pattern. This will then have an adverse affect on the pulse sequences which are required with the use of respiratory triggering.

## PULSE SEQUENCES

This section focuses on the pulse sequences used, their weightings and image planes, and most importantly, validates the reasons for using the parameters to attain maximum diagnostic information. It should be noted that these imaging tools vary from one clinical centre to another [2, 8, 35] and that there are numerous valid reasons for such differences. These may include the preference of the reporting radiologist, adhering to an already established protocol which may be a part of an ongoing prospective study; pulse sequences available from a particular manufacturer, and pulse sequences and technical capabilities available or inherent to a particular software operating platform. The following discussion relates to pulse sequences available on the GE 1.5 Tesla twin speed magnetic resonance (MR) scanner using the HDx operating platform (General Electric Medical Systems, Wisconsin, USA).

### 1. Three Plane Localiser

Always commence with a T1-weighted three plane localiser as this sequence provides low spatial resolution images demonstrating anatomy for orientation purposes. These images are thus used for identifying the initial required anatomical structures for subsequent planning or prescription of the diagnostically proper pulse sequences. This sequence should be acquired with the patient in suspended expiration so that these low resolution images are not further degraded by respiratory motion artifacts.

### 2. Axial 2D FIESTA (Fat Suppressed)

**Figure 1:**
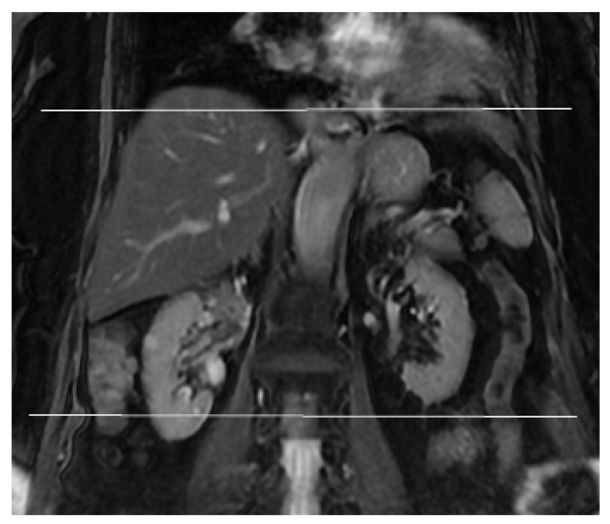
Scanning range prescription for the axial 2D FIESTA Fat Suppressed series.

**Figure 2:**
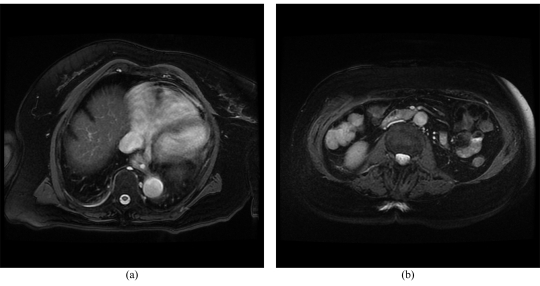
The (a) superior and (b) inferior respective slices of the axial images acquired with 2D FIESTA Fat Suppression.

The purpose of this sequence is to obtain imaging of the hepatic ducts, biliary tree and pancreatic duct in the transverse plane. Fat suppression improves conspicuity of solid lesions and also minimises phase ghosting artefacts from subcutaneous and intraperitoneal fat. This is of greater importance in respiratory triggered sequences [8]. The transverse or axial plane is the most common and therefore familiar of all the imaging planes and it is an orientation which we can easily recognise. By varying the required technical factors a balance of acceptable image quality and scan time can be achieved. Typically, the scan time achievable is approximately between fifteen and twenty seconds. From recent clinical experience, the authors find that most patients are able to hold their breath for this duration regardless of their presenting pathology. The FIESTA pulse sequence provides excellent contrast differentiation of the fluid-filled structures (ducts and gall bladder) with the surrounding anatomy (liver and pancreatic tissue) which includes fat suppression [2]. This sequence serves to demonstrate the gall bladder, the cystic duct and the hepatic and pancreatic ducts. The first prescribed slice should be almost at the most superior aspect of the liver to ensure that the majority of the right and left hepatic ducts are captured. The most inferior prescribed slice should be located into the lumen of the duodenum to ensure that the sphincter of Oddi is captured as well as any variation in the location of the union of the pancreatic duct. These two reference points are indicated in Figure 1, with their corresponding axial images depicted in Figure 2 (a) and (b). This scanning range, plane and sequence-weighting is designed to provide coverage and assessment of the entire biliary tree ducts, the main pancreatic duct and to determine the location of any biliary stones (which appear as hypo-intense) and strictures [4]-5,9,11-12,33]. A relatively high receive bandwidth (RBW) is used to minimise the echo spacing [34]. This serves two main purposes. Firstly, it allows the designated number of prescribed slices to be acquired at an overall shorter scan time. Secondly, it minimises distortion type artefacts such as those arising from metal surgical clips from prior surgery. Additionally, if the RBW had not been as wide, it would not have allowed the same number of prescribed slices scanned with a shorter acquisition time. The down side to an increased receive bandwidth is that it captures a greater amount of noise relative to the signal. However, to the naked eye, this may neither be noticeable nor hinder the image quality for diagnostic reporting purposes.

#### Variation from the norm: possible modification of protocols

As indicated, one of the easiest ways to manipulate acquisition time would be to adjust the RBW. If the patient is large and additional slices need to be prescribed for anatomical coverage, then the RBW can be increased to accommodate for this. The downside is that this will increase the noise content inherent within the image and adversely affect the signal-to-noise ratio (SNR). However, it can be argued that this level of increased noise may not degrade the image significantly to adversely impinge upon the diagnostic quality for the reporting radiologist. An increased RBW also reduces the echo spacing and can thus minimise the image appearance of susceptibility artefacts (such as arising from metal surgical clips). Acquisition times of approximately twenty seconds can be achieved and approximately twenty slices can be prescribed, with the following parameters: a RBW of approximately 85 kilohertz, a slice thickness of 8 millimeters, a spacing of 2 millimeters and a matrix of 256 x 256.

### 3. Coronal 2D FIESTA (Fat Suppressed)

**Figure 3:**
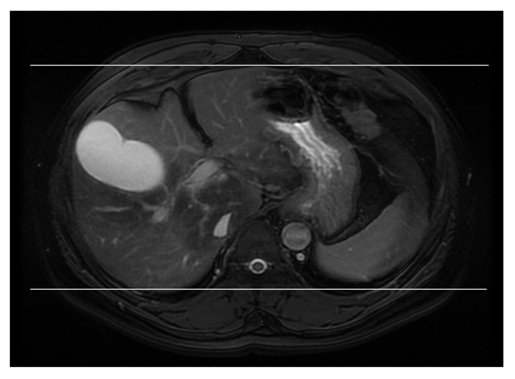
Scanning range prescription for the Coronal 2D FIESTA Fat Suppressed Sequence.

**Figure 4:**
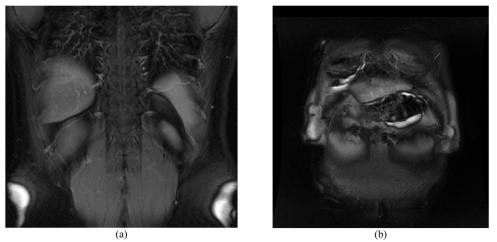
The (a) posterior and (b) anterior respective slices from the scanning range prescription for the Coronal 2D FIESTA Fat Suppressed sequence.

**Figure 5:**
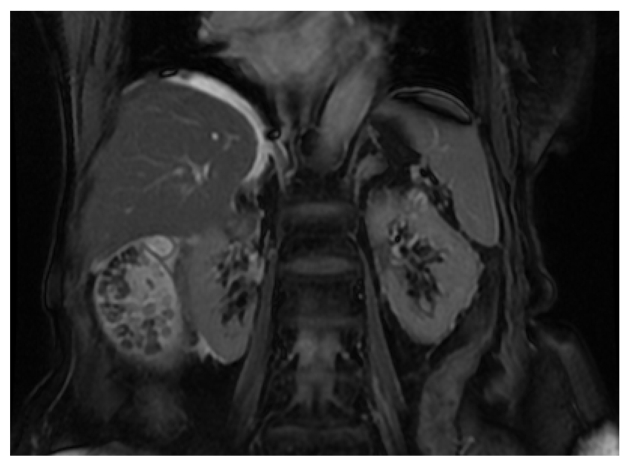
Coronal 2D FIESTA Fat Suppressed image demonstrating multiple stones within the gall bladder.

The reasons for performing the coronal sequence are exactly the same as for the axial series, but another view of the relevant anatomy is obtained. In particular, this plane is useful in adding assessment value to the condition of the CBD, cystic duct, hepatic ducts and the gall bladder; with pathology affecting the ampulla of Vater particularly well noted [4]. The scanning range should have the prescribed slices commencing within the lumen of the duodenum (to visualise any biliary fluid passing through the sphincter of Oddi) and ending at almost the most anterior surface of the liver (to ensure that the intra hepatic ducts are included). The start and end slice prescription is indicated in figure 3 and their resulting coronal images are shown in Figure 4(a) and (b). This scanning range should also provide coverage for the pancreas. However, it is wise to confirm the inclusion of pancreas by scrolling through the axial images with the overlying prescribed coronal slices to ensure that no anomalies or pathology has directed or positioned the pancreas out of this scanning range. Figure 5 is an example of a coronal 2D FIESTA fat suppressed image demonstrating multiple stones within the gall bladder.

#### Variation from the norm: possible modification of protocols

The factors concerning the RBW in the *Coronal 2D FIESTA* is applicable to all sequences. Since this sequence is acquired in the coronal plane, ensure that all precautions have been taken with the field-of-view (FOV) so that phase wrap does not occur. It is likely that a banding or moiré pattern [34] artifact will appear at the corners of the image, particularly at the shoulder and hip regions. This artifact is common on gradient echo-based sequences with a large FOV and the artifact will almost always occur at the periphery of the FOV, in particular at the corners of the image [34]. If the patient’s arms, for example, are in contact with the magnet bore, this would mean that anatomy from outside the FOV can produce a signal that then enters the FOV. Furthermore, the banding, or black and white, effect is due to inhomogeneity of signal being in and out of phase [34]. The most immediate action to take is to ensure that the patient’s anatomy is not in contact with the magnet bore. Contact with the magnet bore also has potential consequences for heating and skin burns. Therefore, ensure that thermal resistant material is placed between the patient and the magnet bore. Otherwise, an alternative pulse sequence can be considered; most likely a spin echo-based sequence with factors altered to offer comparable signal contrast such as to emphasise the fluid-filled ducts and gallbladder.

### 4. Axial T2-Weighted FRFSE Respiratory Triggered

The success of this sequence is a direct result of how well the patient has been prepared and how closely they are able to follow and maintain the correct breathing instructions. Being a T2-weighted sequence, this sequence is valuable for aiding characterisation of extraductal (contained within the liver and pancreas) solid and cystic masses and provides supplementary information regarding fluid within the ducts and any other pathologically associated fluid collection [12]. The scanning range for this sequence is as per the axial 2D FIESTA (fat suppressed) pulse sequence described above.

The purpose of this respiratory triggered sequence is to acquire images of the biliary tree with improved spatial resolution, while maintaining an acceptable level of contrast resolution [8] that is similar to the fat suppressed FIESTA sequence. Some studies have demonstrated that respiratory triggered sequences such as this can demonstrate spatial resolution greater than that achievable with standard breath-hold sequences [37]. To achieve this, the MRI system must have dedicated gradient coils, associated hardware and dedicated software to allow for parallel imaging, and multi-channel receiver (surface) coils to be used. Compared to non-parallel imaging techniques, parallel imaging may have an inherently lower signal-to-noise ratio value [32, 37]. However, the two main advantages that make this sequence justifiable are the improved imaging times [32, 34, 36-37] that come with parallel imaging techniques and the highly prominent contrast demonstrated between the fluid-filled ducts and the background tissue [39].

**Figure 6:**
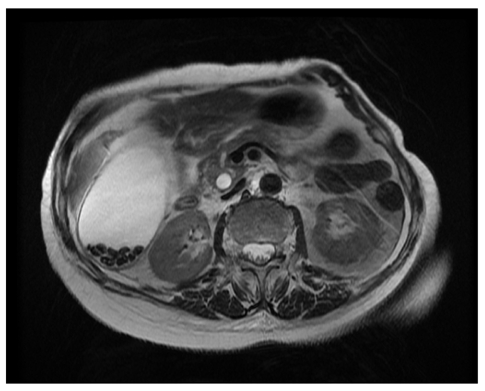
An axial T2 weighted FRFSE Respiratory Triggered sequence demonstrating multiple stones within the gall bladder and diagnostic quality image sharpness.

Respiratory triggered sequences provide improved overall visualisation of the pancreatobiliary system [37] and in particular the main pancreatic duct [6]. This duct is particularly difficult to image due to its relatively large movement with the patient’s rhythm of respiratory motion and it is this respiratory triggered sequence which may provide the greatest information on the condition and calibre of the main pancreatic duct. In addition, if there is respiratory motion along the phase direction, then phase mismapping will result [34, 38, 40]. Therefore, it is imperative that respiratory motion is maintained by the patient in a constant rhythm, and that respiratory monitoring and triggering are performed accurately. Figure 6 demonstrates an axial T2 weighted FRFSE respiratory triggered image showing multiple stones within the gall bladder. It also serves to demonstrate the diagnostic quality of the sequence and overall image sharpness.

#### Variation from the norm: possible modification of protocols

This sequence becomes difficult to perform if the patient has shallow breathing, an irregular breathing pattern or if the bellows are not positioned correctly. Respiratory triggering synchronises the radiofrequency excitation pulse with a phase point of the patient’s respiratory motion [34, 38]. This implies that each prescribed slice should be acquired at the identical point of the respiration cycle. There are two inherent and interlinked challenges of this method: the overall scan time and resulting image contrast may be affected because the repetition time (TR) is determined by the patient’s breathing pattern, that is, either quick or slow. If the patient is capable of breathing in a regular fashion, then this should be encouraged [34, 38]. Factors that may need to be adjusted can be found in the Gating Screen of the operator’s console. The most important parameters are the number of Respiratory Intervals, the Trigger Point, the Trigger Window and the Inter-sequence Delay. The Respiratory Intervals allows the radiographer to select the maximum number of breaths that the patient can take for the system to generate image data from one prescribed slice, therefore the greater this value, the longer the overall scan time will be [34, 38]. The Trigger Point allows the radiographer to determine where along the ascending part of the respiratory waveform peak the data acquisition should take place; and this is usually expressed as a percentage value. If a patient is taking shallow breaths, then a lower value (for example 10% above the trough or baseline) may be suitable, whereas if a patient has comparatively deeper and regular breaths, then a trigger point of 30% to 40% may be more appropriate. The Inter-sequence Delay is a time delay that is added to the end of the TR. This is done so that the radiofrequency excitation pulse that is delivered to commence the pulse sequence will actually coincide with the patient’s breathing cycle and thus minimise phase mismapping [34, 38]. Besides adding time to the overall scan period, this time delay also needs to be added because a patient’s breath cycle is longer than the TR of the sequence. The option available for this is usually 'minimum' or 'even space'. The authors recommend 'minimum' as this is an appropriate balance between achieving a respectable overall scan time (of under four minutes) and synchronising the commencement of the pulse sequence with the patient’s breathing cycle in order to minimise phase mismapping.

### 5. Coronal Oblique 3 Slab MRCP

The underlying concept is to image fluid within the ducts while suppressing signal from non-fluid structures [8]. The main aim of this classic MRCP sequence is to demonstrate ductal fluid as hyperintense while filling defects, such as those caused by stones, are displayed as hypointense [12]. Traditionally, a set of radially oriented thick slab MRCP images were obtained and may still be the case in many centres [44, 47]. It has been somewhat successful and so it is understandable that centres continue to use this approach. This may be of benefit when anatomical structures are difficult to identify on axial images (perhaps due to prior surgery or congenital anomalies) or because of an advanced stage of pathology which results in severe distortion of the relevant anatomical structures. However, the authors believe that this approach, if used for every case, may falsely lead to an oversimplification of the MRCP procedure. It may even be considered as a novice approach since it is not targeted directly at the anatomical structures specific to the biliary tree. An approach which is directed more at the anatomy of the biliary tree, would be to obtain three specific images aimed at the CBD, cystic duct and the pancreatic duct. This procedure is described as follows.

**Figure 7:**
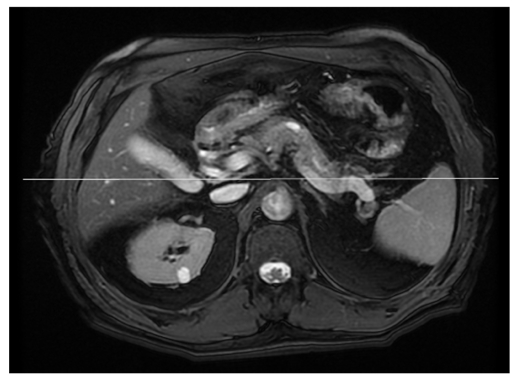
First slice is prescribed coronal, directly through the CBD.

Firstly, from the axial 2D FIESTA F/S images, identify the CBD. This should be easy provided that the patient has fasted adequately, the CBD is filled with bile and fat suppression has worked successfully on the axial 2D FIESTA sequence. Prescribe the first slice straight through the CBD in a coronal fashion as depicted in figure 7.

**Figure 8:**
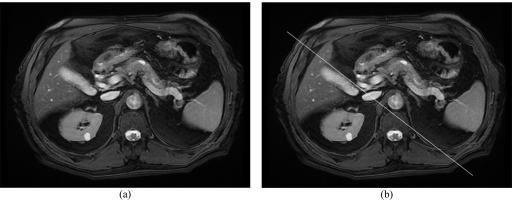
(a) Clearly demonstrates the cystic duct and (b) indicates the second prescribed slice parallel to the cystic duct.

Next, the second slice is prescribed parallel to the cystic duct. Identification of the cystic duct is easier to achieve when the gall bladder is still in situ. Once again, if the patient has fasted the gall bladder should be easily identifiable (except in pathologies such as cystic fibrosis where the gall bladder is invariably contracted and thick walled [54]). The cystic duct connects the neck of the gall bladder to the CBD, therefore the second slice is prescribed as closely as possible along this line, also indicated in figure 8(a) and (b).

If the patient has had the gall bladder surgically resected, there may still be a segment of the cystic duct remaining. This is where an element of difficulty is introduced. From the authors’ experience, the best, most accepted and safest approach is to locate the point along the hepatic duct where the cystic duct forms its union with the CBD. If the junction of the hepatic and CBD cannot be clearly identified, then the next likely solution would be to identify the junction or the region where the first, fourth and fifth liver segments meet. At this point or at the junction of the hepatic and bile ducts, prescribe a slice approximately 45 degrees to the para-coronal plane (used to prescribe the first slice). Bearing in mind that the gall bladder lies on the postero-inferior surface of the right lobe below the porta hepatis and the quadrate lobe; this angle or any variation to this, should be approximate to the angle that the cystic duct makes with the neck of the gall bladder.

**Figure 9:**
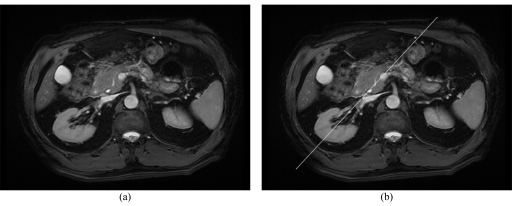
(a) demonstrates a pancreatic head that is pathologic and enlarged. Note that the course of the main pancreatic duct through the head of pancreas is not ideally straight. (b) Indicates how the third slice is prescribed parallel to the main pancreatic duct through the head of pancreas.

The third slice needs to be prescribed parallel through the pancreatic duct along the head of pancreas, figure 9(a) and (b). A normal pancreatic duct has a diameter of 2-3 mm within the head of pancreas. The pancreatic duct needs to be identified on an axial image and the best axial sequence for this will either be the axial 2D FIESTA (fat suppressed) or the axial T2 respiratory triggered. Therefore, it would be advisable to review images from both of these sequences to determine which slice best demonstrates the pancreatic duct.

In recent years, depending on the clinical protocol, secretin is used to help dilate the pancreatic duct. Studies have demonstrated that the intravenous administration of secretin has the effect of allowing the main pancreatic duct to fill with fluid and therefore become more readily identifiable [41-44] while another study has claimed success with the use of intravenous morphine [19]. Time is yet to determine whether these approaches will be routinely performed in the broad clinical setting.

#### Variation from the norm: possible modification of protocols

As this overall acquisition takes several seconds to perform, just about all patients should be compliant to allow this to be successful. However, depending on patient size, one may need to alter the FOV accordingly. Also, there may be software teething on some operating platforms such that all three prescribed slices may not be able to be acquired within the one series; therefore each prescribed slice may need to be its own series, that is, only one slice per series.

### 6. Para Coronal 3D MCRP Respiratory Triggered

This is also a heavily T2-weighted sequence, but acquired as a 3D volume [20]. The main purpose for this approach is to capture a 3D perspective of the biliary tree, and with appropriate software, permit the observer to rotate the volume representation of the biliary tree in order to view its intricacies from practically limitless angles. This is valuable in providing detail in relation to the appearance and calibre of the ducts – remember always that what one has imaged is fluid within the ducts, and thus only providing information of the internal aspect and condition of the lumen. The axial images performed earlier can provide higher quality information pertaining to the duct wall itself.

**Figure 10:**
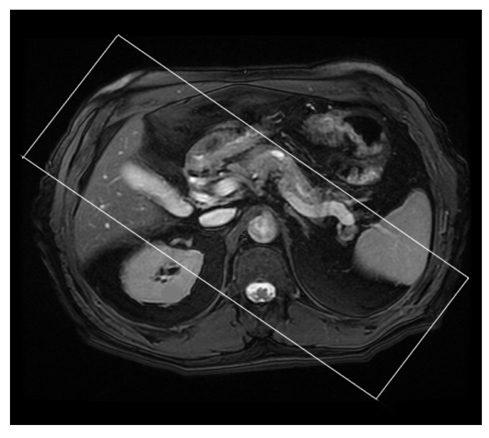
Volume prescription for the Para-coronal 3D MRCP Respiratory Triggered sequence. Ensure that you will capture the biliary tree, gall bladder and the main pancreatic duct. You may need to scroll through the axial series images to check this before commencing the 3D acquisition.

This volume is positioned to capture the entire biliary tree. The para-coronal angle used would be identical to that mentioned in the Coronal Oblique 3D Slab MRCP sequence; that is, the volume is centred to and along the cystic duct, and the volume expanded to include the entire components comprising the biliary tree. This is demonstrated in figure 10. It is important to include saturation pulses immediately adjacent to all boundaries of the imaging volume in order to minimise artefacts originating from both respiratory and physiological motion from degrading the data within the imaging volume.

Once the volume data is acquired, a maximum intensity projection (MIP) data set is generated [4, 16]. Following this, projections are defined at fifteen-degree intervals laterally to a complete 360-degree rotation and also along the superior–inferior axis, once again at fifteen degrees to a completed 360-degree rotation. Therefore, there should be twenty-four (360 divided by 15 equals 24) projections in the lateral axis rotation and a further twenty-four projections in the superior–inferior direction. Of course, if there is a particular area or focus of interest, which would most commonly involve a junction of two ducts (such as at the union of the cystic duct to the hepatic duct, or where the pancreatic and CBD unite), then additional projections that best demonstrate the area of interest are warranted.

It is also prudent to submit the source images to the radiologist for review and reporting. These source images provide greater spatial resolution and can best demonstrate small filling defects and strictures of the pancreatic duct [4, 10, 12, 23]. Depending on their location, stones as small as two millimetres are capable of being detected and projections from a variety of angles may also be of use in such instances [33].

Since spatial resolution can be degraded because of volume averaging effects, as is well noted with 3D acquisitions, this leads to the next discussion on axial thin slices.

#### Variation from the norm: possible modification of protocols

With this sequence, the radiographer must be aware of possible phase wrap artifacts, so before the sequence begins, the FOV must be appropriate. In addition, due to the saturation bands encompassing the imaging volume, they must be carefully positioned for two main reasons: firstly, so that they can minimise physiological and respiratory motion artifacts from degrading the image volume and secondly, so that they do not inadvertently suppress signal within the imaging volume. All the gating and respiratory parameters discussed in the axial T2-Weighted FRFSE Respiratory Triggered sequence also apply here.

### 7. Axial Thin Slices T2-Weighted

**Figure 11:**
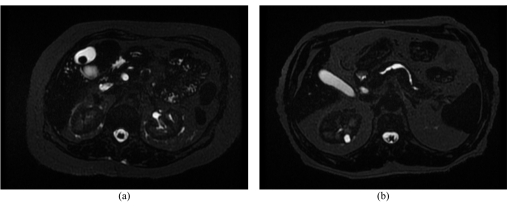
(a) and (b) are of different patients - They are axial Thin Slice T2-Weighted images demonstrating a filling defect (representing a gall stone) (a) within the gall bladder and (b) calibre irregularity of the main pancreatic duct.

The main purpose of this sequence is to acquire biliary tree duct detail with improved spatial resolution such that small dimension pathology can be detected [7] as thick slices tend to obscure small filing defects [33]. The thinner the slice is, the greater the spatial resolution. However, SNR is sacrificed for this [34, 38, 40]. Figure 11 provides image examples of this sequence.

Scanning range once again is from the upper aspect of the liver to the duodenum. Depending on the patient’s body habitus, this may need to be performed over a number of breath-hold cycles. An approach such as this will ensure that there is sufficient overlap of anatomy and the entire biliary tree is scanned. This is also aided by performing each sequence with expiratory breath-hold. As discussed earlier, expiratory breath-hold should provide better consistency for overlapping acquisitions as compared with inspiratory breath-holding. In the timing screen, there should be an option called “resps. before pause” which allows the radiographer to determine the number of respiratory cycles (or breaths) per acquisition. This sequence should have about thirty-five prescribed slices of four millimetre thickness with a spacing of one millimetre. This can be performed over nine acquisitions with four respirations (within each acquisition) before pausing. This should take a total scan time of approximately two minutes and fifteen seconds; equating to one hundred and thirty-seven seconds. One hundred and thirty-seven seconds divided by nine acquisitions equals approximately fifteen seconds. Therefore, each acquisition takes fifteen seconds and will cover four slices. The fifteen-second value should also be displayed in the timing screen.

#### Variation from the norm: possible modification of protocols

The number of respirations before pausing should be selected according to the patient’s capabilities to perform expiratory breath-holds. For example, a patient with associated liver pathology (such as tumour or cirrhosis) or obstructive jaundice or ascites may not be able to hold their breath for fifteen seconds. Therefore, it would be more prudent to offer such patients the equivalent of two respiratory cycles before pausing the sequence; which means that the patient would only need to hold the breath for a more achievable duration of approximately eight seconds.

### 8. “Dynamic” Coronal MRCP

One of the main limitations of the MRCP/MRI examination of the biliary tree and associated organs is that only static images can be obtained [33]. Whereas, with the diagnostic ERCP procedure, the biliary tree can be filled with a contrast medium, and its drainage recorded and observed in real-time with an image intensifier.

**Figure 12:**
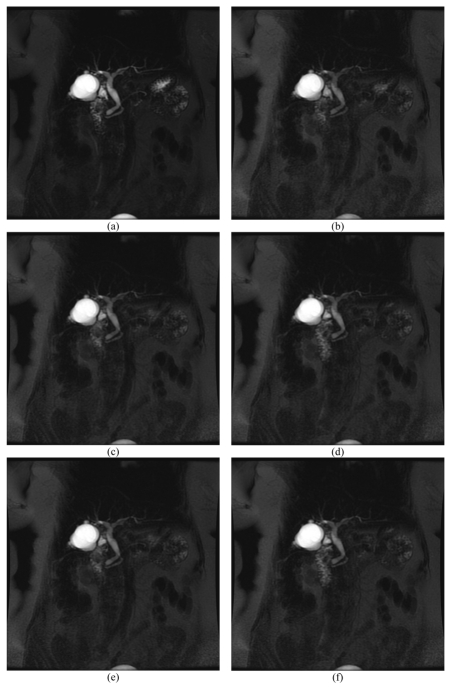
These series of images are of the “Dynamic” Coronal MRCP. Note the movement of the common duct and the fluid drainage into the duodenum that has been captured.

This limitation can be somewhat addressed with the dynamic coronal MRCP sequence. Simply prescribe a slice in the coronal plane directly through the level of the CBD as prescribed earlier in the Coronal Oblique 3 Slab MRCP sequence. Next, instruct the patient to hold a breath on suspended expiration and repeat this sequence six times, thereby acquiring six images at the same location. This is achieved by either clicking on the mouse button to scan, or by pressing the scan button on the keyboard six times. The resulting images should demonstrate fluid drainage into the duodenum or obstruction or strictures along the CBD. Such images are provided in Figure 12 (a) and (f) or alternatively, the video clip (Video 1) generated from these acquired images can be viewed.

**Video 1:** This video clip has been created from Figure 12 to demonstrate the ‘dynamic’ concept of this technique

**Figure 13:**
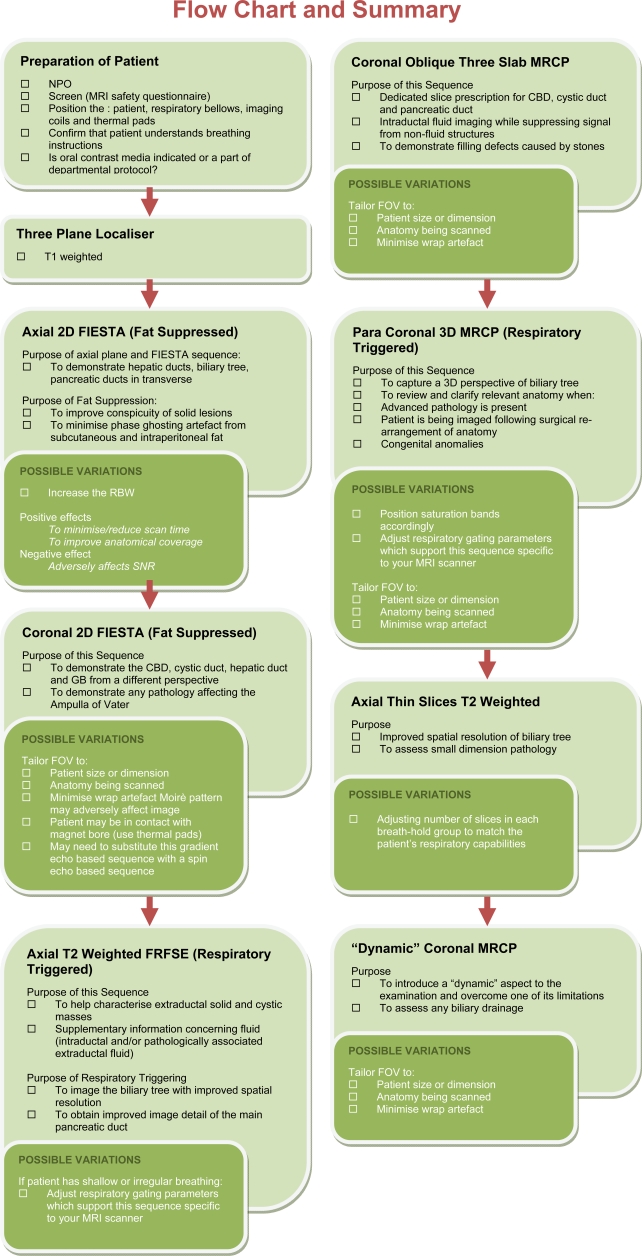
Flow chart, including check-list, for the MRI/MRCP examination. This flow chart is a synopsis of the sequences for the MRCP/MRI procedure. The chart can also be printed and used as a check-list.

#### Variation from the norm: possible modification of protocols

As this final sequence takes a combined imaging time of only a few seconds, there may be very few possibilities for sequence modification as all patients should be capable of complying with this series. The radiographer needs to ensure that the FOV is set correctly so that no phase wrap artifact occurs and that the slice thickness is substantial enough (such as twenty-five millimetres) so that sufficient contrast resolution becomes inherent within the resulting image.

## DISCUSSION>

The use of MRI and MRCP to assess the biliary tree and related organs such as the liver and pancreas is now well established. Numerous publications [2, 20, 25, 45-46] report on the sensitivity, specificity, positive and negative predictive value of the MRCP/MRI assessment of the biliary tree, while others report on its diagnostic accuracy [20, 24]. These studies offer some degree of comparison with the diagnostic component of the ERCP examination. However, these reported values vary greatly and are more often reflective of the pathology being assessed, the stage (early onset or late) at which the pathology is imaged, and the size, dimension or extent of the pathology. These values are also a function of the capabilities and features (hardware and software) of the MRI system used, as well as the expertise of the MRI radiographer.

Although MRCP does not require the administration of contrast media, the inclusion of MRI examination in conjunction with MRCP may necessitate the use of contrast media. One recent trend is the implementation of renal function test prior to the intravenous administration of MRI contrast media in order to identify patients that may be potentially at risk of developing nephrogenic systemic fibrosis (NSF). In the MRCP/MRI setting, the use of contrast media is seen as an alternative to T2-weighted sequences [8], and Gadolinium-based contrast media has proven itself to provide characterisation between benign and malignant tumours; although dynamic acquisitions provide greater detection of hepatic lesions than non-enhanced acquisitions. Kim *et al*. successfully demonstrated that by adding a T1-weighted, contrast-enhanced sequence to the MRCP/MRI examination, malignant biliary strictures were better visualised [20].

There is no doubt that technology is advancing rapidly to help generate higher quality images to further enhance the standard of the MRI/MRCP examination. Emerging trends include the use of automated-type software programmes [5, 37]. In addition, the administration of secretin (2, 7, 41-44) has been used in the assessment of main pancreatic duct and pancreatic pathology. Intravenous morphine is also useful in improving distention of the biliary and pancreatic ducts as it reduces fluid outflow at the ampulla of Vater, which increases intraluminal pressure [29]. Further studies may be required to establish their validity as routine practice. High magnetic field strength at 3.0 T, shows increase in the SNR with improved visualisation of biliary and pancreatic ducts [48]. However, in some instances, the use of endoscopic ultrasound is gaining popularity as an alternative to the ERCP [25] and MRCP as it provides a higher sensitivity in identifying causes of CBD obstruction compared with MRCP [49].

Novel and innovative techniques have also been published. For example, pineapple juice (which has the effect of decreasing T2 signal intensity) has been used as a negative oral contrast agent to improve visualisation of the ampulla of Vater, the CBD and the common hepatic duct [50] by minimising signals from the stomach and duodenum detracting from the biliary and pancreatic ducts. This study purported that pineapple juice may be used as an alternative to the commercially distributed agent, Ferumoxsil.

What is certain is that the MRCP/MRI examination is firmly grounded in the clinical setting and has now become an examination that a MRI radiographer must perform on a very regular basis. It is used in the evaluation of gall stones, infection and inflammation, and malignant and benign tumours [[Bibr R4]-5, 7, 9, 10-12, 33]. A 2005 study by Shanmugam *et al*. [25] has claimed that the MRCP may become the new gold standard due to its high sensitivity and specificity for choledocholithiasis, and may even convincingly replace the diagnostic ERCP in the coming years [7, 10] . Although transabdominal ultrasound remains the initial imaging modality for the biliary system [9], MRCP, spiral computed tomography and endoscopic ultrasound are now essential components to be carefully considered in the diagnostic work-up [51] for patients.

## CONCLUSION

The advantages offered by MRCP/MRI include: no ionising radiation; no or relatively low invasiveness [10] (depending on the administration of contrast media); no risk of induced pancreatitis [25] or other idiopathic treatment complications [2,16,24,52] such as cholangitis due to contrast media retention in patients with advanced biliary tract stenosis; no risk of morbidity or mortality (provided that the patient is correctly screened and is safe to enter the MRI environment and no contrast material is used); and it is less costly compared to ERCP. If patients are presented with the option of the two diagnostic tests (MRCP/MRI or diagnostic ERCP), then patient preference for a more conservative and less invasive approach may further lead to increased bias towards MRCP/MRI [24]. Over the last decade, as the role of MRCP/MRI has rapidly strengthened, there has been a noticeable and corresponding decline in the diagnostic ERCP examination, with fewer and more complex therapeutic ERCP procedures being the norm. This decline in ERCP has implications on ERCP training and practice [53], an issue which is beyond the scope of this paper. Financial benefits of this have previously been discussed. The authors believe that the advantages offered by MRCP/MRI examination will ensure that the MRCP/MRI will be used as a definitive diagnostic tool for assessing biliary and pancreatic ducts as well as a screening tool for determining if patients need to undergo surgical intervention, or if patients can benefit from the therapeutic solutions offered through ERCP.

Thus, radiographers must be aware of the role that the MRCP/MRI examination can play in the overall medical management of patients today. As such, it is critical that MRI radiographers must understand and perform this examination to the highest possible standard.
